# Literary Notices

**Published:** 1872-11

**Authors:** 


### LITERARY NOTICES.

LIPPINCOTT'S MAGAZINE, for December, is, as usual, well filled with entertaining and instructive articles, some of which are finely illustrated. The table of contents is very rich and attractive. Among these we observe an article entitled, "Nurse and Patient," from the pen of that distinguished American physician, S. Wier Mitchell, M. D.,—an article that will prove instructive to all classes of readers. Lippincott's Magazine is elevated in tone, is exceedingly well edited and beautifully printed. It is a pleasure to read it. Price $4 per annum.

APPLETON'S JOURNAL, always fresh and spicy, is a general favorite. With a large corps of writers, and copiously illustrated, its weekly visits are welcomed by hundreds of readers everywhere. Edited with great ability, and unsurpassed in typographical appearance, it is a treat and luxury to the reader to secure it. Price, $4 per annum.

HEARTH AND HOME, the family illustrated weekly, is unsurpassed. Elevated in moral tone, pure in sentiment and faithful in all things, it becomes both a teacher and a friend in the family circle. Besides the large amount of reading matter, news, etc., its stories are excellent, and its illustrations are "legion." A beautiful "Chromo" will be given each subscriber for 1873. Orange Judd & New York. Price, $4 per annum.

SCIENTIFIC AMERICAN.—This is a weekly journal of practical information, Art, Science, Mechanics, Chemistry and manufactures. It is an invaluable journal to the inventor and one and all cannot fail to be read with pleasure. Many of the most useful inventions of the day are illustrated and described in its pages. Munn & Co., No. 37, Park Row, New York. Price, $3 per annum.

THE ILLUSTRATED RECORD AND REPOSITORY—Is one of the largest, best and finest pictorial papers published. It is pure, high-toned, and contains the choicest of American and Forcign literature. Specimen copy, ten cents. Send for it—address, Illustrated Record and Repository, P. O. Box 2141, New York.

KIND WORDS—Is a charming little paper, finely illustrated and devoted to the religious training of children. It is conducted by that accomplished christian gentleman, Rev. S. Boykin, Memphis, Tennessee. Parents, send for Kind words. It will do your children good and they will thank you for it.

